# Septic shock complicated by disseminated herpes simplex virus-1 infection: a case report

**DOI:** 10.1186/s13256-021-02985-1

**Published:** 2021-08-08

**Authors:** Amélie Boquet, Guillaume Boulay, Etienne Hautin, Nicolas Mottard

**Affiliations:** 1 Department of Anesthesiology and Critical Care, Clinique de la Sauvegarde, RAMSAY Santé, Lyon, France; 2 Service de Réanimation, Clinique de la Sauvegarde, 480 Avenue Ben Gourion, 69009 Lyon, France

**Keywords:** Herpes simplex virus, Septic shock, Multiorgan failure

## Abstract

**Background:**

Herpes virus remains dormant in human cells and could reactivate under immunosuppressed conditions, such as prolonged critical illnesses. The phenomenon of viral replication during intensive care is well known, even in patients without a history of immunosuppression, but it usually does not have a clinical impact. Systemic reactivation leads to viral DNA in blood. It remains unclear whether this replication is a marker of morbimortality or a true pathogenic process. Therefore, it is unclear what medical treatment is most appropriate for simple replication. In organ damage suspected to be induced by herpes virus, there is no consensus on the most appropriate treatment duration. Here, we report a rarely described case of multiorgan failure implicating herpes simplex virus and discuss its treatment.

**Case report:**

A 53-year-old Caucasian immunosuppressed woman was admitted to the intensive care unit for septic shock. She presented pneumonia due to *Klebsiella pneumoniae*. Two weeks after admission, she showed multiorgan failure with acute respiratory distress syndrome and circulation failure. She had digestive and cutaneous lesions typical of herpes simplex virus 1. Blood and respiratory polymerase chain reaction was strongly herpes simplex virus-1 positive. No other bacteria, fungi, or viruses were found. The evolution was rapidly favorable after the initiation of antiviral treatment. Treatment was stopped after 3 weeks of well-conducted antiviral therapy. Curative-dose treatment was interrupted despite continuous strongly positive blood polymerase chain reaction results. In this context, prophylactic treatment was continued.

**Conclusion:**

We report an exceptional presentation of multiorgan failure in the intensive care unit due to herpes simplex virus-1. The diagnosis was made based on typical herpes simplex virus-1 visceral lesions and the absence of other responsible microorganisms. Intense viral replication is a key diagnostic element. There is no consensus regarding the most appropriate treatment duration, but such decisions should not be based on blood polymerase chain reaction.

## Introduction

Viral reactivation affects critically ill individuals and even patients without prior immune deficiency. It remains unclear whether such viral infection is actually pathogenic or simply a severity factor. Herpes virus organ damage in the intensive care unit (ICU) is mainly induced by pneumonia. As described in our case, disseminated viral infection is rarely observed in the ICU setting. In this context, there is an indication for antiviral therapy. There are, however, no guidelines for treatment duration.

## Case report

A 53-year-old Caucasian woman was admitted to the intensive care unit for septic shock secondary to hypoxic pneumonia induced by *Klebsiella pneumoniae*. Medical history included anti-signal recognition particle (SRP) autoimmune myositis, treatment with corticosteroids and methotrexate, and active smoking.

Her ICU stay was initially due to severe septic shock, and she required high-dose vasopressor amines and intravascular volume therapy > 30 ml/kg. Probabilistic antibiotic therapy started with cefotaxime (2 g/8 hours) and rovamycine (3 million IU/8 hours). The condition was then complicated by multiorgan failure, including septic acute kidney injury requiring renal replacement therapy (RRT), hepatic cytolysis for 20 cycles without liver dysfunction, and acute respiratory failure requiring intubation, sedation, paralysis and two sessions of prone positioning. Amoxicillin/clavulanic acid (2 g/250 mg/8 hours) was then initiated and adapted to the antibiogram, achieving improvement. Norepinephrine was withdrawn, and hepatic cytolysis resolved. Sedation was terminated, enabling the patient to awaken under spontaneous ventilation with inspiratory assistance.

On day 14, the patient again showed hemodynamic and respiratory deterioration.

Sedation was reinitiated under controlled ventilation with high-dose noradrenaline (up to 2 µg/kg/minute) to achieve hemodynamic stability. Kidney failure did not resolve, and RRT was continued. Probabilistic antibiotic therapy was initiated with imipenem (1 g/6 hours), amikacin (25 mg/kg/day), vancomycin (2 g/day), and fluconazole (400 mg/day). Compared with the previous scan, thoracic CT revealed significant degradation in the numerous bilateral pulmonary condensation sites. The presentation corresponded to severe acute respiratory distress syndrome. Two days after this further deterioration, the patient presented upper digestive tract hemorrhage requiring emergency gastroscopy, which revealed esophageal ulcerations suggestive of viral esophagitis. More or less concomitantly, there were intraoral, labial, vulvar, perianal vesicles and cutaneous vesicles, mainly on the anterior side of the lower limbs and forearms and, less densely, on the back and trunk. During the following days, episodes of rectorrhagia required rectoscopy, which also found ulcerated and bullous lesions with an aspect similar to a geographic map in the lower rectum. Corneal examination with fluorescein screened for herpetic keratitis and was negative. Contrast-enhanced brain CT was performed to screen for complicated herpetic meningoencephalitis, as clinical neurologic examination was precluded by the sedation ensuring ventilatory compliance; the results were normal, with no signs of cerebral edema, intracranial hypertension, or encephalitis. Lumbar puncture was not performed owing to curative-dose anticoagulation therapy linked to a right jugular thrombosis, and positive findings would not have modified the treatment.

Biologically, there was leukocyte elevation (mainly polymorphonuclear neutrophils) at 32 giga/liter. CRP and procalcitonin were elevated at 131 mg/L and 4.2 µg/L, respectively. There was further hepatic cytolysis; aspartate aminotransferase (AST) was 4412 IU/L with conserved factor V. Lactate was elevated at 3.7 mmol/L. Initial bronchoalveolar lavage prior to antibiotic therapy showed no bacteria or fungi, but PCR was highly positive for herpes simplex virus 1 (HSV-1) and slightly positive for HHV-6 and EBV. PCR in total blood and cutaneous vesicles was also highly positive for HSV-1. Digestive endoscopy was performed in a hemorrhagic context; no biopsies were performed.

Meningeal dose acyclovir (10 mg/kg/day) was initiated for the onset of disseminated vesicles and continued for 21 days. Second, low-dose prophylactic treatment was continued to prevent recurrence in this immunosuppressed context.

Clinical progression became favorable after 72 hours of well-conducted antiviral treatment, with hemodynamic improvement allowing withdrawal of vasopressors, followed by respiratory improvement and rapid regression of hepatic cytolysis. Mucosal and cutaneous vesicles disappeared after 3 weeks. After several episodes of severe rectorrhagia requiring hemostatic rectoscopy, digestive complications regressed within 1 month. Control PCR on total blood was performed at 1 month post-onset (1 week after treatment termination) and was still highly positive, although with greater dilution. The control was therefore repeated at 2 months, with no clinical relapse, and was still positive, although again with greater dilution.

The patient was discharged from the ICU after 2 months of intensive care. Withdrawal and rehabilitation were hindered by muscular impairment, probably due to in-ICU neuromyopathy and decompensation of chronic muscular pathology.

## Discussion

The seroprevalence of herpes simplex virus is estimated at 70% in developed countries [1]. Viral sepsis accounts for less than 1% of septic episodes requiring stays in the intensive care unit in these countries. The viruses most frequently implicated in ICU pneumonia [other than coronavirus disease 2019 (COVID-19)] are rhinovirus, parainfluenza virus, human metapneumovirus, and influenza virus [2].

There have been many reports of HSV pneumonia [3], and some cases of fulminant hepatitis [4] and sepsis [5] implicate HSV in immunocompetent and immunosuppressed patients. The present case is a rare description of HSV-1-induced septic shock causing multiorgan failure. No other possible responsible viral, bacterial, or fungal microorganisms were identified in the patient’s lungs or blood; HSV-1 replication was very elevated, and associated involvement was diffuse.

In ICUs, the incidence of HSV-1 reactivation in septic shock is high, at 32–68% [6]. This may be due to inevitable immune modulation to combat the primary pathogen, even in patients who are immunocompetent at admission [7]. It is important here to know whether HSV-1 isolated from the lower respiratory tract is a marker of severity or an actual pathogen implicated in pulmonary disease [8]. Viral reactivation in the ICU is associated with higher rates of mortality and complications (ventilation time, hospital stay) [6]. However, it remains to be shown whether this is a marker of severity or actually causal, and it is thus unclear whether antiviral treatment will be beneficial. In patients with VAP not responding to antibiotic treatment and with high HSV load, Schuierer *et al*. [3] showed that acyclovir treatment was associated with a significantly longer time to death in the ICU, reduced hazard ratio for ICU death, and improvement in morbidity (mechanical ventilation time, vasopressor dose). There is, however, at present, no consensus on such treatment. The present patient showed diffuse disease implicating HSV-1, and treatment was unavoidable.

Treatment was started 48 hours after the onset of hemodynamic and respiratory deterioration at meningeal dose for 21 days, as meningoencephalitis could not be excluded. Deterioration continued despite 48 hours of wide-spectrum probabilistic antibiotic therapy. After 48 hours, the vasopressor dose was strongly reduced, with withdrawal at 72 hours. Respiration also improved within a few days, and hepatic cytolysis regressed. The cytolysis could not be definitively attributed to viral hepatitis but might have been secondary to circulation failure. There are no guidelines on antiviral treatment time in such cases. We tried relying on reduction of plasma PCR HSV-1, but this remained strongly positive after 1 month, despite 3 weeks of well-conducted treatment. Nevertheless, the patient had by then normalized clinically, and it was decided not to reintroduce treatment on the basis of the biology findings; PCR was positive 2 months after symptom onset.

Luyt *et a*l. reported that PCR on bronchoalveolar lavage was more effective than viral culture in diagnosing bronchopneumonitis [8]. This is a highly sensitive molecular biology technique for screening viral DNA but does not represent viral pathogenesis. Blood clearance of viral DNA is not known, and blood PCR is not a good guide for treatment. In light of the present results, blood PCR does not seem to be a reliable method for determining the appropriate duration for antiviral treatment or even to assess the effectiveness of treatment. In cases like the one presented herein, low-dose prophylaxis should be continued for patients with symptomatic HSV infection.

## Conclusions

Herein, we described an exceptional situation of HSV-induced septic shock with disseminated pulmonary, cutaneous, and digestive disease. In such cases, it is unclear what antiviral treatment duration is most appropriate; molecular research via PCR does not seem to be a reliable guide.

## Data Availability

Not applicable.

## Abbreviations

DNA: Deoxyribonucleic acid
HSV: Herpes simplex virus
ICU: Intensive care unit
PCR: Polymerase chain reaction
RRT: Renal replacement therapy
CT: Computed tomography
CRP: C-reactive protein
HHV-6: Human herpes virus 6
EBV: Epstein–Barr virus
